# Spinal cord stimulators and radiotherapy: First case report and practice guidelines

**DOI:** 10.1186/1748-717X-6-143

**Published:** 2011-10-25

**Authors:** Lorraine Walsh, Daipayan Guha, Thomas G Purdie, Philippe Bedard, Alexandra Easson, Fei-Fei Liu, Mojgan Hodaie

**Affiliations:** 1 Radiation Medicine Program, Princess Margaret Hospital, University Health Network, 610 University Avenue, Toronto M5G2M9, Ontario, Canada; 2 Department of Radiation Oncology, University of Toronto, 610 University Avenue, Toronto M5G2M9, Ontario, Canada; 3 Department of Neurosurgery, University Health Network, 610 University Avenue, Toronto, M5G2M9, Ontario, Canada; 4 Division of Medical Oncology, Princess Margaret Hospital, University Health Network, 610 University Avenue, Toronto M5G2M9, Ontario, Canada; 5 Division of Surgical Oncology, Princess Margaret Hospital, University Health Network, 610 University Avenue, Toronto M5G2M9, Ontario, Canada

**Keywords:** radiotherapy, spinal cord stimulator, pacemaker, electronic device

## Abstract

Spinal cord stimulators (SCS) are a well-recognised treatment modality in the management of a number of chronic neuropathic pain conditions, particularly failed back syndrome and radiculopathies. The implantable pulse generator (IPG) component of the SCS is designed and operates in a similar fashion to that of a cardiac pacemaker. The IPG consists of an electrical generator, lithium battery, transmitter/receiver and a minicomputer. When stimulated, it generates pulsed electrical signals which stimulate the dorsal columns of the spinal cord, thus alleviating pain. Analogous to a cardiac pacemaker, it can be potentially damaged by ionising radiation from a linear accelerator, in patients undergoing radiotherapy. Herein we report our clinical management of the first reported case of a patient requiring adjuvant breast radiotherapy who had a SCS *in situ*. We also provide useful practical recommendations on the management of this scenario within a radiation oncology department.

## Background

Radiotherapy (RT) is a major cancer treatment modality, applied to approximately 60% of patients at some point in their natural history for curative and palliative intent [1]. However, RT can interfere with and potentially damage implanted electronic devices such as cardiac pacemakers or implanted cardioverter-defibrillators (ICD) [2]. Given the increasing use of these devices for a broad range of medical applications, recommendations have been developed regarding the safe delivery of RT with such devices *in-situ *[3,4]. Safety guidelines do not yet exist however, for implanted electronic spinal cord stimulators (SCS).

Spinal cord neuromodulation using implantable electrodes placed over the dorsal columns in the epidural space can be an effective strategy for the control of severe, longstanding, neuropathic pain [5,6]. The SCS system consists of 3 components: an electrode array which is implanted in the epidural space overlying the dorsal columns of the spinal cord; an implantable pulse generator (IPG) which consists of an electrical generator, battery, transmitter/receiver and a minicomputer, which is placed beneath the skin and controlled transcutaneously by the patient; and insulating wiring connecting the electrodes to the IPG. These devices are an important treatment modality for chronic neuropathic pain conditions refractory to conservative management, including complex regional pain syndrome, radiculopathies, failed back syndrome, phantom limb pain, and post-herpetic neuralgia. They are increasingly applied to other conditions including intractable angina and ischemic pain secondary to peripheral vascular disease, though long-term efficacy remains undetermined [7].

Surgical techniques for SCS implantation are largely similar regardless of indication and are typically performed in two stages. One to four leads are placed in the epidural space in the cervical, thoracic or lumbar regions as appropriate, either percutaneously or *via *a small laminotomy. The SCS can also be placed to cover exiting nerve roots in the epidural space, in addition to or instead of posterior placement over dorsal columns. The leads are inserted under local anesthesia with sedation, following which they are connected to an external connector lead and controller that allows the patient to manipulate the device once ambulating. The majority of patients are trialled as out-patients. Less commonly patients can remain in hospital for 2-5 days after the procedure during which time pain level and functional capacity improvements are assessed. The system is deemed suitable for insertion (stage 2) if there is adequate pain relief (typically greater than 50% pain relief) and lack or minimal side effects of stimulation. The IPG is then implanted into a subcutaneous pocket and connected to the leads. Typical IPG locations include the gluteal and flank regions, with sub-clavicular and abdominal wall placement performed less frequently [8].

The IPG closely resembles and operates similarly to a cardiac pacemaker, generating pulsed electrical signals which stimulate the dorsal columns. The amplitude (intensity), frequency, and width of the stimulating electrical signals can be adjusted for the desired effect. The IPG contains a battery similar to that of a cardiac pacemaker, typically a single lithium thionyl chloride cell or lithium-iodine battery. The terminals of the battery cell are connected to the input terminals of the voltage regulator, which provides power to the logic and control section of the IPG. The logic and control section includes a microprocessor and controls the programmable functions of the device via a crystal oscillator (Rutecki: United States Patent, Number 5330515, July 1994). When irradiated however, the IPG could be potentially damaged by the ionizing radiation itself, or by electromagnetic interference generated from the linear accelerator, in the same way that ionising radiation can damage a cardiac pacemaker or ICD.

Herein, we present the first reported case of a patient with a SCS *in-situ*, who required adjuvant RT for breast cancer. We outline our management plan in ensuring the safe delivery of radiation for this patient, and suggest recommendations for the management of this uncommon, but potentially serious clinical scenario.

## Clinical Case

A 46 year old peri-menopausal woman, with no known breast cancer risk factors, self-detected a left breast mass. Digital mammography identified a 10 cm mass occupying the left breast. Biopsy identified a grade 3 ductal carcinoma *in-situ *(DCIS). Fine needle aspiration of a left axillary node identified invasive ductal carcinoma (IDC). Metastatic work-up was negative for distant disease. Clinical stage was hence T3N1M0.

A left-sided mastectomy and axillary lymph node dissection was performed. Pathological analysis identified multi-focal grade 3 IDC, the largest focus measuring 0.4 cm, on an 8.6 cm background of grade 3 DCIS, with extensive lymphovascular space invasion. Two of fourteen lymph nodes were positive for metastatic disease. The closest surgical margin to invasive disease was 1 mm; 2 mm for the DCIS. Oestrogen and progesterone receptors were negative, but HER-2/neu was positive. Pathological stage was T1aN1aM0 (multi-focal).

Multidisciplinary tumor board discussion recommended adjuvant loco-regional RT, in view of extensive grade 3 DCIS, node positivity, and close surgical margins. Adjuvant systemic chemotherapy plus trastuzumab was also recommended.

**Figure 1:**
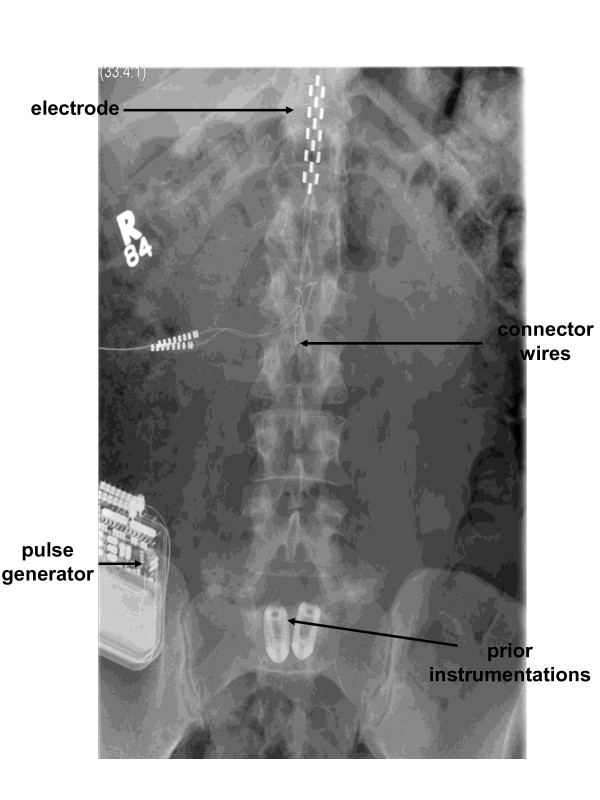
**An AP X-ray demonstrating position of the epidural spinal cord stimulation electrode, connector wires, and pulse generator located in the right lower quadrant**. The patient's prior instrumentation in the lumbo-sacral area is also visualized.

Prior to her cancer diagnosis, this patient had an 8-year history of chronic back pain, following an acute posterior L4-L5 disc herniation, requiring urgent lumbar spine decompression. Her pain was further aggravated following a motor vehicle accident. She underwent spinal fusion for L5-S1 spondylolisthesis in 2005 (Figure 1). Her lack of relief of chronic leg pain eventually resulted in the diagnosis of failed back surgery syndrome (FBSS). Given the severity of her neuropathic radicular pain, the lack of adequate response to conservative measures, and the interference of pain with her daily life, she subsequently had a SCS inserted (Figure 1). She obtained a good response with decreased neuropathic pain and increased mobility. The patient-controlled device allows her to "dial" the stimulus to effect, within a pre-arranged stimulus range. She can also turn the device off, if necessary.

Following initial consultation with Radiation Oncology, a thorough literature search was initiated regarding the delivery of RT to patients with a SCS, which yielded no reports. We sought advice from her consultant neurosurgeon (MH), who specializes in SCS placement. The device manufacturer Medtronic^® ^was contacted and device specifications obtained (Prime Advanced, Model: 37702, Serial number: NKL706993H). The verbal recommendation from the manufacturer was to avoid direct irradiation of the device. She consented for RT, following a discussion highlighting the benefits and risks of treatment, including the measures to be undertaken to protect her SCS.

### Radiotherapy Planning

The patient underwent a simulation-CT scan in a standard treatment position. Given our concern regarding the effects of RT on the IPG in her right iliac fossa, the entire device was imaged on the simulation-CT. By contouring the IPG, an estimation of the received dose by the IPG during RT delivery was calculated, giving a dose of 0.7 cGy per fraction. The device was 22 cm from the inferio-medial radiation field edge. An IMRT technique to encompass the left chest wall and supra-clavicular fossa was used. Dose to organs at risk was minimized according to internationally recognised dose constraints.

### Radiotherapy Delivery

Treatment was delivered with 6 MV photons, administering daily 200 cGy fractions to a total dose of 5000 cGy over 5 weeks (Elekta Synergy). During RT delivery, the patient turned off her SCS. Dosimetric readings were obtained on three separate occasions during RT using metal oxide-silicon semiconductor field effect transistor (MOSFET) diodes. These were placed on the skin surface in build-up material on the patient's right iliac fossa (opposite side of treatment field). Readings were also obtained on the left iliac fossa, in the event that the IPG had been originally placed in that location (same side as treatment field) (Table 1). Treatment was completed as planned. The maximum acute toxicity was grade 2 radiation dermatitis.

**Table 1:** Semiconductor diode surface measurements at the pulse generator location (patient's right side, contralateral to radiation fields)

Diode Readings: Right Iliac Fossa	Diode Readings: Left Iliac Fossa
Reading 1: 0.00 cGy	Reading 1: 0.32 cGy ± 0.3 cGy ^†^
Reading 2: 0.00 cGy	Reading 2: 0.00 cGy
Reading 3: 1.11 cGy ± 0.32 cGy*	Reading 3: 0.33 cGy ± 0.2 cGy

Readings were also performed on patient's left side (ipsilateral to radiation fields).

*Had the pulse generator received this dose at each fraction this would have equated to a total dose of 27.7 cGy ± 8.1 cGy over a total radiation schedule of 50 Gy in 25 fractions of 2 Gy each (= 0.5% of total dose).

^† ^Had the pulse generator received this dose at each fraction, this would have equated to a total dose of 8 cGy ± 7.5 cGy over a total radiation schedule of 50 Gy in 25 fractions of 2 Gy each (= 0.16% of total dose).

## Discussion and Recommendations

Apart from this case report, specific investigations regarding the impact of RT on SCS systems have not been performed to date, perhaps partly accounted for by how infrequently this clinical scenario arises. However, given that the range of indications for SCS continues to expand, it is imperative that guidelines exist regarding the safe delivery of RT to a patient with a SCS *in situ*. As we have described above, the IPG component of the SCS operates similarly to that of a cardiac pacemaker, and therefore it is possible to extrapolate from the evidence of cardiac devices to understand how RT can be damaging to implanted electronic devices; hence determining what safety procedures should be followed.

The radiation effects on cardiac devices have been widely studied, with safety recommendations provided [4,9-12]. The potential damaging effects are two-fold: a) interference caused by electromagnetic interference (EMI) from the linear-accelerator; and b) effects resulting from direct radiation beams. Damage is dependent on the total delivered dose, the type of radiation used, and the device specifications. Damage can be caused when the device is on or off, can be transient, or can result in permanent re-arrangements of the atoms within the semiconductor crystal [13].

Last *et al *described how ionizing radiation can affect cardiac pacemaker function [14]. When directly irradiated, electron-hole pairs are created in the silicon and silicon-dioxide semiconductor materials. Once the radiation is off, the electron-hole pairs rapidly recombine, and the electrons leave quickly by flowing to the metal or semiconductor within the oxide. This can result in either transient or permanent changes within the pacemaker. The holes being relatively immobile remain in the valence band, and are attracted by structural defects, which can lead to an accumulation of trapped positive charges, also deleterious to the pacemaker. The trapped charges in the oxide can in turn produce an alteration in the current-voltage characteristics of the device, potentially causing aberrant electrical pathways.

Venselaar *et al *examined the effects of ionizing radiation on pacemakers irradiated in a cobalt-60 beam, and the influence of EMI by two linear-accelerators [15]. In the cobalt-60 beam, two pacemakers demonstrated a decrease in pulse repetition frequency when irradiated to therapeutic dose levels, with two others demonstrating pacing failure at 97 Gy and 147 Gy, respectively. When determining EMI sensitivity, an inhibition of one pacemaker pulse occurred when one linear accelerator was turned on and off.

The American Association of Physicists in Medicine (AAPM) issued guidelines in 1994 for the safe irradiation of patients with pacemakers [3]. In contrast to Venselaar *et al*, this report stated that EMI effects were both negligible and transient when modern contemporary linear-accelerators were used; hence EMI was not a cause for concern. However, it acknowledged that the harmful effects of the radiation itself remained.

Mouton *et al *irradiated 96 different pacemakers, with different dose rates and fraction sizes [16]; 14.6% demonstrated a clinically relevant failure under 5 Gy, with a 6% failure rate even below 2 Gy. Hurkmans *et al *investigated the newest generation of ICDs and cardiac pacemakers [9,10]. Eleven modern, unused ICDs were irradiated to 20 Gy in seven fractions. Subsequent irradiation was to a definite point of failure or to 120 Gy. Whilst most withstood irradiation to ≥ 80Gy, some displayed serious malfunctions, including complete loss of function in four devices at doses as low as 2.5 Gy. Inappropriate shock delivery occurred in four instances. No EMI was recorded.

Given that no formal safety guidelines were available to us in our management of this patient, we initially calculated the expected dose to be received by the IPG at the time of RT planning, giving an estimated total dose of 18.4 cGy for all 25 fractions. In addition, diode readings were taken on 2 separate occasions with 4 measurements on each occasion. This approach, in addition to the safety procedures recommended for cardiac pacemakers, forms the basis of the practice guidelines we have developed for the safe delivery of RT to patients with a SCS *in situ*. It is important to recognise however, that there are limitations to these guidelines. We have no measurement of the maximum tolerated dose of the device, and the 5 Gy limit is the extrapolation from the use of cardiac pacemakers, where the consequences of damage can be life-threatening. It is certainly possible that a higher dose limit could be recommended for the SCS system; however in the absence of any dosimetric data from our own institution or from the available literature supporting a higher dose limit, we would currently recommend a 5 Gy limit. It is also possible that different SCS systems with varying device specifications have different radiation tolerances, as is the case with cardiac pacemakers and ICDs. It is also important to state that if the IPG is within the radiation field, it might be more feasible to actually remove it with or without the connecting leads, but leaving the epidural electrode in place. These can be reinserted later with minimal morbidity. Accepting these limitations, we would recommend adherence to the guidelines as outlined in Table 2. These guidelines provide general safety recommendations, in addition to advocating a step-by-step approach to the simulation, planning and delivery of radiation to patients with a SCS *in situ*.

**Table 2:** Recommendations for patients with a SCS system *in-situ *requiring radiation

General Recommendations	1. All patients with SCS should be identified prior to treatment planning, and appropriate action taken to limit and minimize the exposure of the device to radiation. 2. Adopt a multidisciplinary approach and seek neurosurgical involvement prior to treatment delivery. 3. Obtain detailed product specification from the device manufacturer (e.g. model and serial numbers, expected battery life). 4. Obtain assurance that the SCS is in proper working order prior to treatment planning and delivery. 5. Obtain full written informed consent from the patient
**Simulation and Planning Recommendations**	1. Ensure the patient is simulated in a comfortable and reproducible treatment position. 2. Do not perform MRI simulation (or other MRI imaging without contacting the device manufacturer) with a SCS *in-situ* 3. At the time of CT simulation, the radiation therapist must ensure that the entire device is visible on the simulation images if it is within 30 cm of the proposed radiation fields. 4. During treatment planning, calculate the estimated dose which the device will receive.

**Treatment Delivery Recommendations**	1. The pulse generator should be placed at least 1 cm outside of the direct therapy beam during treatment delivery. For IMRT plans, all segments should be checked to ensure no beam passes through the device. 2. The patient is asked to turn the device to 'off-mode' during the actual treatment delivery. It is acknowledged however, that damage can occur to electronic devices during radiation whether powered on or off. 3. Dosimetric measurements should be taken on 3 separate occasions by placing diodes at the skin surface over the pulse generator under suitable build-up conditions. Extrapolating from the dose limit recommendations for cardiac pacemakers, the recommended total dose limits to the pulse generator should be less than 5 Gy.

## Conclusion

Whilst the consequences of RT on the IPG may not be as serious as those on pacemakers or ICDs, and while further dosimetric data regarding the radiation tolerance of such SCS systems remain to be generated, in the interim, both the planning and delivery of RT to those with an implanted SCS should follow a methodical approach to ensure its safe delivery. This will help to avoid any unnecessary damage to the device, which could result in significant pain and morbidity for the patient, and the possible requirement to replace such a device.

## Consent

Written informed consent was obtained from the patient for publication of this case report and accompanying images. A copy of the written consent is available for review by the Editor-in-Chief of this journal.

## Competing interests

The authors declare that they have no competing interests.

## Authors' contributions

LW participated in the radiotherapy management of this patient and drafted the manuscript. DP contributed the neurosurgical components of the manuscript. TP performed the radiotherapy planning, and dosimetric measurements and calculations for this patient. PB contributed the chemotherapy components of the manuscript. AE contributed the surgical oncology components of the manuscript. FFL was the primary radiation oncologist involved in this case, prescribed the radiotherapy, and contributed to the manuscript. MH was the primary neurosurgeon involved in the management of this case and contributed to the manuscript. All authors read and approved the final manuscript.
